# Unnoticed for 14 years: an asymptomatic forgotten common bile duct stent – a case report

**DOI:** 10.1097/MS9.0000000000003078

**Published:** 2025-11-07

**Authors:** Swsan A. Elsharif, Mustafa A.M. Elamin, Ahmed Rafei, Elaf M. Abdelraheem, Abdelmounem E. Abdo

**Affiliations:** a Faculty of Medicine, University of Khartoum, Khartoum, Sudan; b Faculty of Medicine, Al Neelain University, Khartoum, Sudan; c Research Department, National Center for Gastrointestinal and Liver Diseases, Khartoum, Sudan; d National Center for Gastrointestinal and Liver Diseases, Khartoum, Sudan

**Keywords:** asymptomatic stent, choledocholithiasis, ERCP, forgotten biliary stent

## Abstract

**Introduction:**

Choledocholithiasis is the presence of gallstones within the common bile duct (CBD), affecting 10–20% of gallbladder stone patients. Endoscopic retrograde cholangiopancreatography (ERCP) with stent insertion is a common treatment of CBD obstruction and stenosis to maintain duct patency, with stents typically replaced within 3–6 months. However, prolonged stent presence can lead to complications such as pancreatitis and cholangitis. We report a rare case of a neglected CBD stent left in situ for 14 years without complications.

**Case presentation:**

A 77-year-old female with controlled hypertension and diabetes presented to the hospital for a routine check-up. Physical examination was unremarkable except for mild scleral jaundice. She had a history of obstructive jaundice treated with ERCP and stent placement in 2010 but missed follow-ups for stent replacement. Laboratory tests revealed mildly elevated total bilirubin and low serum albumin. An abdominal ultrasound showed gallbladder sludge and a mildly dilated CBD. ERCP confirmed a dilated CBD and common hepatic duct stricture. A new plastic stent was placed, with follow-up ERCP scheduled every 6 months.

**Clinical discussion:**

Forgotten biliary stents (FBS) are plastic stents left in place for more than a year. They can cause complications such as cholangitis, jaundice, stent migration, and pancreatitis. Elderly patients are more susceptible due to physical limitations and potentially inadequate follow-up. Our case highlights the unusual presentation of an asymptomatic FBS for 14 years.

**Conclusion:**

This case emphasizes the importance of regular monitoring and timely stent replacement to prevent complications. Effective patient education and adherence to follow-up protocols are crucial in managing biliary stents.

## Introduction

Choledocholithiasis is the presence of gallstones within the common bile duct (CBD). It develops approximately in 10–20% of patients with gallbladder stones.^[1]^ Endoscopic retrograde cholangiopancreatography (ERCP) is a well-established surgical intervention for the management of CBD stones. Following the ERCP procedure, stent insertion is frequently performed to maintain the patency of the duct during the postoperative period.^[2,3]^ A CBD stent constitutes a foreign entity within the body. As anticipated, its prolonged presence is frequently associated with a spectrum of complications, including acute pancreatitis, potential nidus for future CBD stones, acute cholangitis, and uncommon yet life-threatening stent migration^[4]^. Unless emergent of such complications, an ERCP is typically scheduled for stent replacement approximately 3–6 months following initial placement.^[5]^ Considering these adverse prognoses, we report a case of neglected CBD stent in place for 14 years, remarkably demonstrating an absence of symptoms and complications.

**Highlights**

- A 77-year-old Sudanese woman presented with asymptomatic obstructive jaundice due to a forgotten common bile duct (CBD) stent left in place for 14 years.
- Despite being asymptomatic, the patient presented with mild scleral jaundice and was found to have a long-term CBD stent.
- ERCP confirmed CBD dilation and a common hepatic duct stricture. A new stent was placed, with follow-up ERCP scheduled every 6 months.
- The case underscores the need for effective patient education and adherence to follow-up protocols for managing biliary stents.
- The case emphasizes the importance of regular monitoring and timely stent replacement to prevent potential complications associated with forgotten biliary stents.
- Forgotten biliary stents (FBS) can remain asymptomatic for years, highlighting the need for regular monitoring and patient education to prevent complications

This case report is being reported per the SCARE (Surgical CAse REport) guidelines^[15]^.

## Case presentation

A 77-year-old female with a BMI of 27 presented to the hospital for a routine check-up for her well-controlled hypertension and diabetes. Vitals were within the reference ranges. Physical examination revealed no abnormalities except for a mild yellowish discoloration of the sclera of 1-month duration. Her past medical history is significant for obstructive jaundice due to CBD stones, for which she underwent ERCP and a CBD stent was placed in 2010. However, she did not undergo scheduled stent replacement and was lost to follow-up for 14 years. Upon referral to the gastroenterology department for further workup, a thorough abdominal examination was unremarkable, with no tenderness, distention, or organomegaly. The patient had no other complaints of itching, anorexia, nausea, vomiting, or weight loss. Laboratory investigations revealed the following results: alkaline phosphatase – 110.0 U/L, aspartate transaminase – 20.0 U/L, alanine transaminase – 11.0 U/L, total bilirubin – 1.1 mg/dL, direct bilirubin – 0.2 mg/dL, indirect bilirubin – 0.9 mg/dL, total protein – 8.0 g/dL, serum albumin – 2.6 g/dL, and prothrombin time/international normalized ratio – 13.5/1. All other investigations were within normal limits (Table 1).

**Table 1:** The patient’s laboratory findings

Investigations	Results	Reference range
**Complete blood count**		
White cell count (× 10^9^/L)	9.74	4.6–10.2
Red cell count (× 10^6^)	6.38	4.04–6.13
Hemoglobin (g/dL)	16.0	10.4–15.7
Hematocrit (%)	52.1	33–46
Platelets (× 10^9^/L)	229.0	140–450
**Liver profile**		
Total protein (g/dL)	8.0	6.5–8.7
Serum albumin (g/dL)	2.6	3.5–5.3
Total bilirubin (mg/dL)	1.1	Up to 1.1
Direct bilirubin (mg/dL)	0.2	Up to 0.25
Indirect bilirubin (mg/dL)	0.9	Up to 0.75
Alkaline phosphatase (ALP)	110	40–129 U/L
Alanine transaminase (ALT)	11	Up to 34 U/L
Aspartate transaminase (AST)	20	Up to 31 U/L
**Bleeding profile**		
Prothrombin time (PT)	13.5 s	11–19.5 s
INR	1.0	Up to 1.2
**Renal profile**		
Blood urea (mg/dL)	10	10–50
Blood creatinine (mg/dL)	1.1	Up to 1.1
Serum Na (mmol/L)	135	135–145
Serum K (mmol/L)	3.8	3.5–5.0

**Figure 1.**
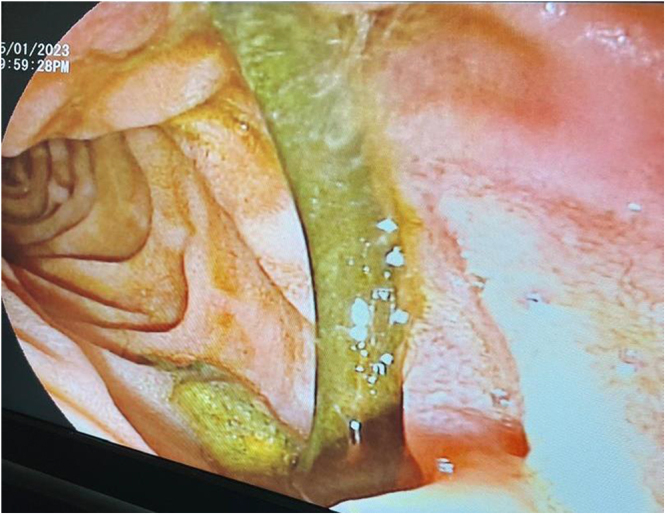
Endoscopic image of the stent in situ.

**Figure 2.**
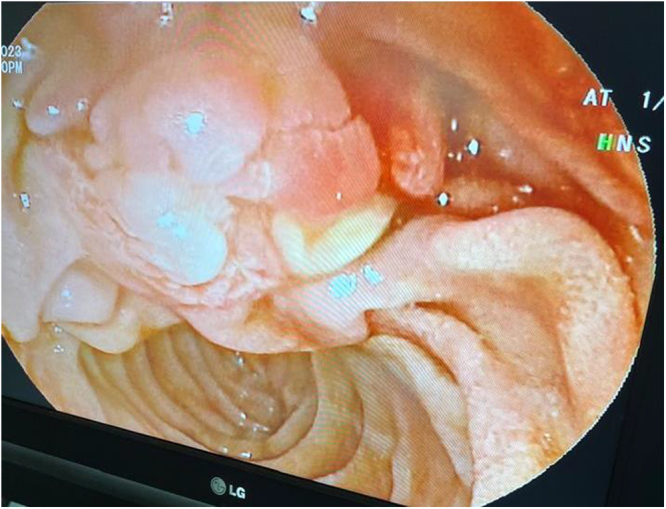
Endoscopic image of the ampulla after stent extraction. The changes observed in the ampulla are due to the long-standing presence of the stent.

**Figure 3.**
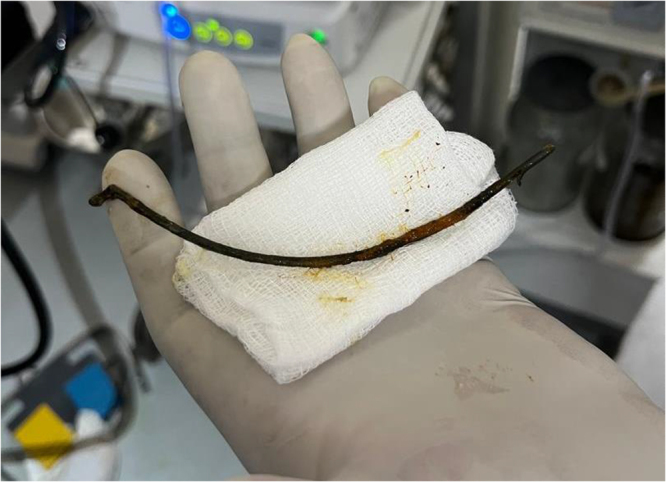
The stent after removal.

An abdominal ultrasound showed gall bladder sludge with no stones or signs of inflammation, and a mildly dilated CBD. Given her medical history, we scheduled an ERCP with a stent replacement. The procedure revealed dilation of CBD and intrahepatic ducts with common hepatic duct stricture. A plastic stent was inserted, resulting in intact bile reflux (Figures 1, 2, and 3). Follow-up ERCP with stent replacement is planned every 6 months.

## Discussion

Endoscopic biliary stenting is used for the treatment of different obstructive biliary diseases, such as benign conditions like choledocholithiasis, biliary stricture, and severe cholangitis, and for the treatment of malignant obstructive jaundice^[6]^. Biliary stents are either plastic or metallic. Plastic stents are used to temporarily relieve obstructions and to maintain bile flow^[7]^. This procedure stabilizes the patient before a subsequent surgical intervention or endoscopic treatment. Definite stenting with metallic stents is recommended for elderly patients with severe comorbidities who are not good candidates for ERCP or surgical intervention^[7]^. The outcome of biliary stenting is good drainage and a low complication rate, but with time, stent occlusion can occur, more often with plastic stents^[8]^. Therefore, plastic biliary stents are recommended to be removed or exchanged within 3–6 months of deployment to avoid infectious complications^[7]^.

Forgotten biliary stents (FBS) are defined as plastic stents that remained in situ for more than 12 months^[9,10]^. The quick relief of symptoms and the endoscopic nature of the procedure may contribute to the instances of FBS. Patient’s lack of awareness, noncompliance, inadequate counseling, and poor documentation may also play a role^[10]^. A study found that the most common reason for FBS reported by patients was that they were not informed about the long-term management of the stent^[9]^. Trainee doctors often forget to counsel patients about stent follow-up and removal, and this in turn leads to this problem^[11]^. Biliary stents are frequently forgotten in elderly patients, as suggested by our case and other reported cases^[5,10]^. This may be explained by the fact that elderly patients face physical impairment, increased morbidity, and dependency, which may prevent them from seeking medical care. In countries like Sudan, financial constraints and a lack of healthcare facilities may also play an important role.

FBS can be asymptomatic and discovered accidentally during routine examination as in the case of our patient and other reported cases^[5]^. It can remain asymptomatic for a long period and then symptoms appear. Patients can present with abdominal pain, nausea and vomiting, fever, itching, and jaundice^[10,11]^.

FBS can cause many complications, mainly cholangitis associated with CBD stones, obstructive jaundice, internal stent migration, and pancreatitis^[5,9]^. It can rarely cause stentolith “de novo formation of biliary stones around the stent”^[9]^. FBS can also remain silent for years without any obvious complications^[12]^.

According to the literature, patients with FBS are either symptomatic with complications, symptomatic without complications, or asymptomatic with complications^[5,10-14]^. Interestingly, our patient was asymptomatic and did not have any complications, and this was a unique presentation.

Symptoms and complications are mainly due to stent obstruction. In benign disease, there is a passage between the CBD and the stent, which allows bile flow if the stent is obstructed. This may explain the absence of symptoms and complications in our patient^[13]^.

Our patient exhibited clinical signs of mild jaundice during a routine follow-up visit. However, the laboratory findings, including serum bilirubin levels from the liver function tests, may not accurately reflect her clinical condition. This discrepancy can be attributed to the current challenges in the healthcare sector following the war and the subsequent collapse of the healthcare system in Sudan. With many government central hospitals unable to perform essential laboratory tests, patients are compelled to seek private laboratory facilities. This often results in substantial delays between clinical evaluation and laboratory testing, potentially leading to misalignments between clinical presentation and laboratory results.

Uncomplicated FBS is usually treated by ERCP with stent removal and re-stenting^[5,11]^. In complicated cases, ERCP typically fails, necessitating surgical intervention. The initial step is often CBD exploration, followed by additional procedures as needed, such as cholecystectomy, choledochoduodenostomy, or hepaticojejunostomy^[10]^.

Forgotten biliary stents constitute a huge health burden, with many serious complications that can be avoided if patients follow the recommended guidelines. Several measures have been proposed to increase patient adherence and compliance. A randomized controlled trial examined the effectiveness of short message service (SMS) on stent removal/exchange and found that patients’ adherence increased to stent removal/exchange after receiving the SMS^[14]^.

There is also promising evidence regarding the effectiveness of biodegradable biliary stents. Although they are still in trial, they will serve as a valuable alternative to conventional stents in patients with poor compliance^[12]^.

In conclusion, this case underscores the importance of diligent follow-up and patient education to prevent complications associated with prolonged stent placement. While our patient did not experience adverse effects, regular monitoring and timely stent replacement are crucial in managing patients with biliary stents to avoid potential complications.

## Data Availability

No additional data are available.
